# Gastro-Jejunostomy

**Published:** 1898-09-24

**Authors:** 


### GASTRO-JEJUNOSTOMY.

The last touch in the technique of gastro-enterostomy seems to have been given by Mr. Lockwood, who, in addition to surrounding the opening between the stomach and the jejunum with a triple row of sutures, has lined it with a layer of mucous membrane, thus not only lessening the likelihood of contraction of the cicatrix which always attaches to operations in which the mucous membrane has been removed from the entire circumference of a canal, but also, which is a far more important thing, protecting the cut surfaces of the mucosa and serosa of the stomach from the corrosive action of the gastric juice. The case which he records[1] was that of a man, aged 54 years, who in 1895 began to have dyspepsia and pain after food. This was soon followed by vomiting of all solid food, and when he was admitted to the Great Northern Central Hospital on January 1st, 1896, there was vomiting also of liquids. Rapid loss of weight had occurred, and a hard, ovoid swelling, the long diameter of which was about 3 in., and the short diameter 2½ in., was easily felt through the thin abdominal wall beneath the eighth right costal cartilage and in the situation of the pylorus. The stomach was dilated, and a succussion splash could be heard and felt. When the abdomen was opened, it was at once evident that the disease was far too extensive to admit of removal in the then exhausted condition of the patient. The front wall of the stomach and the commencement of the jejunum were drawn out of the wound, and being held together were sewn to one another by a row of interrupted silk sutures for about three inches, the line of attachment being parallel with the greater curvature of the stomach, and with the mesenteric edge of the jejunum, and about half an inch from both. This line of sutures only perforated the peritoneum. Next a pair of incisions, each an inch and a half long, were made, one into the stomach and the other into the jejunum, parallel with the line of sutures, and a third of an inch in front of it. These incisions only extended through the peritoneal and muscular coats, and when they had been made the mucous membranes of each organ was separated from the muscular layer for about a third of an inch all round the incision. The posterior edges of the two incisions were then united by a line of interrupted sutures parallel to the first line. The mucous membranes of the stomach and jejunum were then laid open in the same line as the previous incisions, and the posterior edge of the opening in the one organ was attached to the posterior edge of the wound in the other. Then in like manner the anterior edges of the incisions in the mucous membranes were brought together, then the incisions in the muscular and peritoneal layers, and lastly, another line of sutures was inserted through the peritoneum alone, parallel to, and corresponding in front with, the posterior line of sutures by the insertion of which the operation was commenced. The result of these manœuvres was to surround the opening between the stomach and the jejunum by three separate rings of suture, one uniting mucous membranes only, the next the muscular and peritoneal layers, and the outer one the peritoneal layers alone. The operation took a little more than an hour, but in another case the same operation has since been done in forty-five minutes. The result has been admirable. Needless to say, any surgeon who attempts such an operation must be able to sew.

[1] Lancet, Sept 17.

